# Transcriptome and open chromatin analysis reveals the process of myocardial cell development and key pathogenic target proteins in Long QT syndrome type 7

**DOI:** 10.1186/s12967-024-05125-7

**Published:** 2024-03-25

**Authors:** Peipei Chen, Junyu Long, Tianrui Hua, Zhifa Zheng, Ying Xiao, Lianfeng Chen, Kang Yu, Wei Wu, Shuyang Zhang

**Affiliations:** 1 Department of Clinical Nutrition & Health Medicine, Peking Union Medical College Hospital, Chinese Academy of Medical Sciences and Peking Union Medical College, Beijing, China; 2 Department of Liver Surgery, Peking Union Medical College Hospital, Chinese Academy of Medical Sciences and Peking Union Medical College, Beijing, China; 3 Department of Cardiology, Peking Union Medical College Hospital, Chinese Academy of Medical Sciences and Peking Union Medical College, Beijing, 100730 China; 4 Department of Orthopedic Surgery, Peking Union Medical College Hospital, Chinese Academy of Medical Sciences and Peking Union Medical College, Beijing, China; 5 State Key Laboratory of Complex Severe and Rare Diseases, Peking Union Medical College Hospital, Chinese Academy of Medical Sciences and Peking Union Medical College, Beijing, China

**Keywords:** Long QT syndrome type 7, Andersen–Tawil syndrome, Induced pluripotent stem cells, Development and differentiation, Chromatin accessibility

## Abstract

**Objective:**

Long QT syndrome type 7 (Andersen–Tawil syndrome, ATS), which is caused by KCNJ2 gene mutation, often leads to ventricular arrhythmia, periodic paralysis and skeletal malformations. The development, differentiation and electrophysiological maturation of cardiomyocytes (CMs) changes promote the pathophysiology of Long QT syndrome type 7(LQT7). We aimed to specifically reproduce the ATS disease phenotype and study the pathogenic mechanism.

**Methods and results:**

We established a cardiac cell model derived from human induced pluripotent stem cells (hiPSCs) to the phenotypes and electrophysiological function, and the establishment of a human myocardial cell model that specifically reproduces the symptoms of ATS provides a reliable platform for exploring the mechanism of this disease or potential drugs. The spontaneous pulsation rate of myocardial cells in the mutation group was significantly lower than that in the repair CRISPR group, the action potential duration was prolonged, and the Kir2.1 current of the inward rectifier potassium ion channel was decreased, which is consistent with the clinical symptoms of ATS patients. Only ZNF528, a chromatin-accessible TF related to pathogenicity, was continuously regulated beginning from the cardiac mesodermal precursor cell stage (day 4), and continued to be expressed at low levels, which was identified by WGCNA method and verified with ATAC-seq data in the mutation group. Subsequently, it indicated that seven pathways were downregulated (all p < 0.05) by used single sample Gene Set Enrichment Analysis to evaluate the overall regulation of potassium-related pathways enriched in the transcriptome and proteome of late mature CMs. Among them, the three pathways (GO: 0008076, GO: 1990573 and GO: 0030007) containing the mutated gene KCNJ2 is involved that are related to the whole process by which a potassium ion enters the cell via the inward rectifier potassium channel to exert its effect were inhibited. The other four pathways are related to regulation of the potassium transmembrane pathway and sodium:potassium exchange ATPase (p < 0.05). ZNF528 small interfering (si)-RNA was applied to hiPSC-derived cardiomyocytes for CRISPR group to explore changes in potassium ion currents and growth and development related target protein levels that affect disease phenotype. Three consistently downregulated proteins (KCNJ2, CTTN and ATP1B1) associated with pathogenicity were verificated through correlation and intersection analysis.

**Conclusion:**

This study uncovers TFs and target proteins related to electrophysiology and developmental pathogenicity in ATS myocardial cells, obtaining novel targets for potential therapeutic candidate development that does not rely on gene editing.

**Graphical Abstract:**

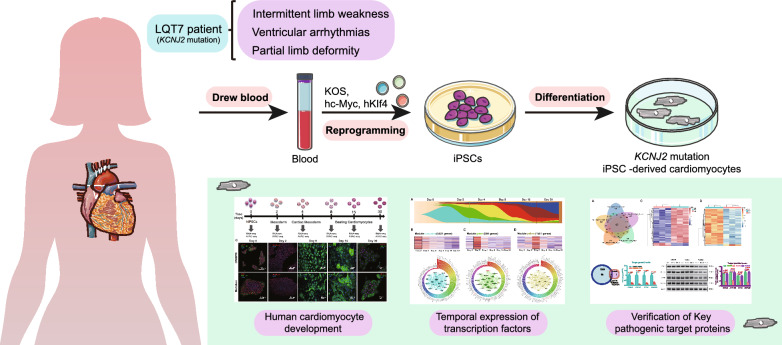

**Supplementary Information:**

The online version contains supplementary material available at 10.1186/s12967-024-05125-7.

## Background

Long QT syndrome type 7 (LQT7), also called Andersen–Tawil syndrome [1], is a rare potassium channel disease involving multiple systems that shows autosomal dominant inheritance or arises from sporadic individual mutation [2, 3]. The prevalence of LQT7 is approximately one in a million [2, 3], and 70% of cases are related to abnormal *KCNJ2* gene function [4]. The inward rectifier potassium channel protein Kir2.1, which encoded by the *KCNJ2* gene is mainly expressed in ventricular muscle, skeletal muscle and brain tissue [5], and plays a crucial role in development. The whole gene knockout mice will have dysplasia after birth and cannot survive for a long time [6]. Therefore, the "typical" clinical triad observed in LQT7 patients consists of QT interval prolongation and ventricular arrhythmia, periodic paralysis and skeletal dysplasia [7]. More than 97% of LQT7 patients have cardiac-related abnormalities, which further lead to serious cardiogenic adverse events [8]. Although inward rectifier potassium channel Kir2.1 may affect the electrophysiological function of myocardial cells [9], the changes in electrophysiological maturation and chromatin accessibility that affect myocardial development and differentiation are unknown. Herein, we established a cardiac cell model derived from human iPSCs to specifically reproduce the ATS disease phenotype and analyze changes of potential transcription factors (TFs) and related target proteins in the transcriptome and open chromatin status at different stages of cardiac development and differentiation. Then, the overall expression of members of potassium ion-related pathways was analyzed, and key target proteins were further screened and verified to help explain the pathogenesis of LQT7.

## Materials and methods

### Sample ethics statement

The current study (Ethics No. JS-1233) received approval from the Ethics Committee of the Peking Union Medical College Hospital, Chinese Academy of Medical Sciences, and Peking Union Medical College (Beijing, China). The investigation conformed to the principles outlined in the Declaration of Helsinki.

### iPSC culture

Peripheral blood was obtained from LQT7 patients and age- and sex-matched control volunteers with no clinical history after informed consent was obtained. We used the mature method [10, 11] to verify the extraction of hematopoietic stem cells from PBMCs to establish iPSCs. Our team has used this method to construct stem cells to study other diseases [12].

### CRISPR/Cas9‑mediated targeted gene correction in iPSCs derived from patient with LQT7

An isogenic gene-corrected control was generated using CRISPR/Cas9-mediated HDR with ssODN, which provided the wild-type template for gene correction. To correct the heterozygous c.199C > T mutation in exon 2 of the *KCNJ2* gene, a mutation-specific sgRNA (target site: ATGTGGGTGAGAAGGGGCAA) was designed using CRISPR, as previously described [13]. sgRNA was generated by in vitro transcription using the GeneArt™ Precision gRNA Synthesis Kit (A29377, Thermo Fisher Scientific, Waltham, MA, USA) according to the manufacturer's instructions. ssODN was designed to contain 123 nt in total, with 83 nt upstream and 40 nt downstream of the Cas9 cut site (5′-gacaggacgaaagccaggcagaagataaccagcatccaccgccagcgaatgtccacacacgtggtgaagatgtctgcgaggtaTcGttgccccttctcacccacattgatgaactgaacattacag-3′). hiPSCs at 80%-85% confluence were harvested with 0.5 mM EDTA. A total of 1.0 × 10^5^ cells were coelectroporated with 400 ng of sgRNA, 2 μg of recombinant Cas9 protein (A36496, TrueCut™ Cas9 Protein v2, Thermo Fisher Scientific, Waltham, MA, USA) and 10 pmol of ssODN using a 10-μl Neon® Tip and a Neon transfection system (MPK5000S, Thermo Fisher Scientific, Waltham, MA, USA) with conditions of 1100 V, 20 ms and 2 pulses, as previously described [14]. Immediately after electroporation, the cells were transferred into one well of a Matrigel-coated 24-well plate and cultured in 500 μl of mTeSR1 medium containing 5 μM Y-27632 (S1049, Selleck, Houston, TX, USA). The medium was changed daily, and the Y-27632 was removed 24 h later. Three days after electroporation, the cells were dispersed at low density into a Matrigel-coated 60-mm dish in mTeSR1 medium with 5 μM Y-27632, which was removed 24 h later. Approximately 14 days later, large colonies were picked, expanded, and analyzed for the desired base correction by Sanger sequencing of an amplicon spanning the target site of *KCNJ2* gene exon 2. A positive clone was selected for further analysis. Genomic DNA was extracted from the iPSCs using a Genomic DNA Extraction Kit (DV811A, Takara, Japan), followed by PCR amplification using a KAPA Taq EXtra HotStart ReadyMix PCR Kit (KK3604, Merck, New Jersey, USA). PCR was performed on a Peltier thermal cycler (PTC-100, Bio-Rad, Hercules, CA, USA) using the following conditions: 94 °C, 3 min; 30 cycles of 94 °C, 25 s; 55 °C, 15 s; 72 °C, 1 min; and 72 °C, 3 min. The PCR product was subsequently sequenced by Sanger sequencing at Shanghai Personal Biotechnology Co., Ltd. (China) to confirm correction of the heterozygous c.199C > T mutation of the *KCNJ2* gene.

### Teratoma assay

For this assay, four-week-old immunocompromised SCID mice (Beijing Vital River Laboratory Animal Technology Co., Ltd., Beijing, China) were used. Cells (2–4 × 10^6^) were subcutaneously injected into the flank area of each mouse. Tumor growth was monitored weekly by palpation. The mice were euthanized when the tumor volume reached ≥ 2 cm^3^ after 8 weeks. All animal experiments were approved by the Animal Ethics Committee of the Peking Union Medical College Hospital.

### iPSC-CM differentiation and treatments

The mutation, CRISPR and control iPSC lines were used in this study. We referred to a well-developed method used in our previous research [12], and CM differentiation was initiated using a monolayer differentiation method with a STEMdiff CM Differentiation Kit (05010, STEMCELL Technologies Inc, British Columbia, Canada) according to the manufacturer's instructions. After the iPSC-CMs had grown to a density of 80%, ZNF528 siRNA (siZNF528) was transfected into CMs using lipofectamine 3000 (Invitrogen L3000015, USA). After co-transfection for 48 h, the cells were used for further experiments. The primer of si-ZNF528 were showed in Additional file 1: Table S1.

### iPSCs- and iPSC-CM-specific protein immunofluorescence staining and confocal microscopy

Pluripotency markers of iPSCs were measured by using a hESC marker panel (1:1000; ab109884, Abcam plc., Cambridge, UK). The structural composition of iPSC-CMs could be identified by the presence of α-actinin and cardiac contractile proteins. So we used antibodies against Brachyury (ab209665, Abcam plc., Cambridge, UK), α-SMA (A5228, Sigma), NKX2.5 and TNNT-2 kit (Thermo Fisher A25973). Stained beating cells and monolayer cells were observed by using laser-scanning confocal microscopy (LSM 710, Carl Zeiss, Jena, Germany), and captured with the Delta Vision OMX SR imaging system.

### Single-cell patch clamp experiment for recording action potentials and quantified current signals

The mature myocardial cells were digested into single cells with digestive enzymes 2 days in advance and adhere to the cover glasses. The whole-cell patch clamp experiment was conducted to measure the Cm with a single beat at 32–35 °C through the EPC-10 patch clamp amplifier (HEKA Electronics, Lambrecht, Germany). The whole-cell Kir2.1 potassium current was recorded with the help of an EPC-10 patch clamp amplifier controlled by PULSE software (HEKA Electronics). At the same time, PatchMaster software (HEKA Electronics) was used to capture data.

### Sample collection for RNA-seq, ATAC-seq and Proteomic analysis

We selected 6 specific time points for collection of the mutation and CRISPR groups using Accutase (A1110501, Thermo Fisher Scientific, Waltham, MA, USA) to perform RNA-seq and ATAC-seq. ATAC-seq was performed as previously reported [15]. Two groups of CMs were collected for further proteome analysis. Quantitative proteomics and mass spectrum analysis were performed by TMT labeling as described in the previous study [16].

### WGCNA and ssGSEA

The package of “WGCNA” within R was used for constructing differentially expressed genes coexpression network and modules [17]. We further evaluated the overall regulation of potassium channel-related pathways at the gene and protein levels via single-sample gene set enrichment analysis (ssGSEA) with the “GSVA” R package [18].

### Expression analysis of target genes and proteins by qPCR and Western blot

The qRT‒PCR and western blot were used to determine the mRNA expression levels of the target proteins. The primers were either obtained from previous studies [19–21] or purchased as a commercial reagent (ORIGENE HP215938) (Additional file 1: Table S1). We chose to isolate total RNA from CMs that exhibited a rate of differentiation greater than 85%, and gene expression was normalized based on the levels of β-actin. Cells with substandard differentiation rates will be discarded. As described in our previous study [12], the collection, detection, and visualized analysis of sample proteins are carried out. Proteins were detected with antibodies specific to β-actin (1:5000, ab8227, Abcam, Cambridge, UK), KCNJ2 (1:1000, ab85492, Abcam, Cambridge, UK), CTTN (1:2000, ab68438, Abcam, Cambridge, UK), ATP1B1 (1:2000, ab193669, Abcam, Cambridge, UK), and ZNF528 (1:1000, PA5-41182, Thermo, Massachusetts, US).

### Resource availability

Further information and requests for resources and reagents should be directed to and will be fulfilled by the first author, Peipei Chen.

### Statistical analysis

Numerical data are shown as the mean ± SD. We used Excel software (Windows 2019, Microsoft, Redmond, US) to compare experimental groups through Student’s t test (two tailed). Differences for which *p* < 0.05 were considered significant.

## Results

### Clinical details and genetic profiles of ATS patients

**Fig. 1:**
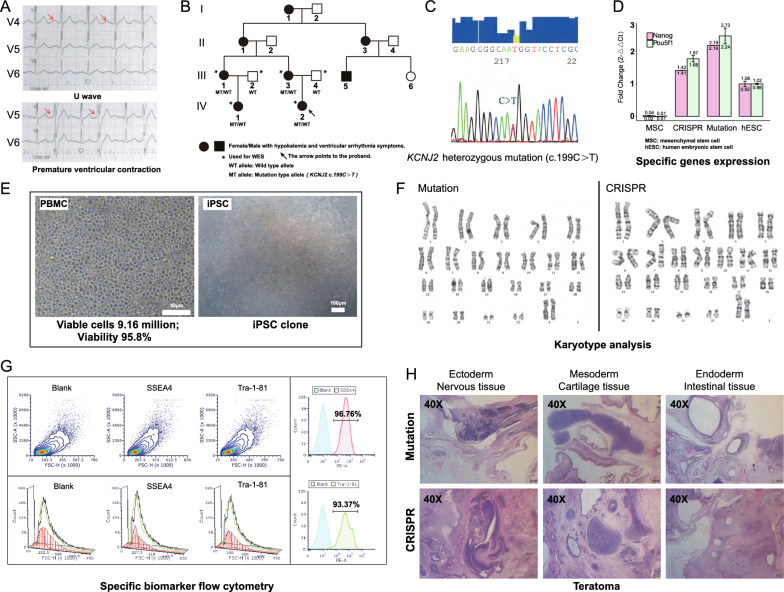
Information on LQT 7 patient and establishment of induced pluripotent stem cells. Images and quantifications are from at least three (n ≥ 3) independent differentiations for each hiPSC lines. **A** Twelve-lead electrocardiography examination of a patient with LQT7. **B** Pedigree of the LQT7 patient in this study. Squares indicate males; circles, females; filled-in symbols, clinically affected individuals; blank symbols, clinically unaffected individuals. **C** Schematic diagram showing the location of the *KCNJ2* mutation, which was verified by Sanger sequencing. **D** iPSC lines showed RNA expression of the pluripotency-related genes POU5F1 and NODAL by RT‒PCR analyzed (MSC n = 3, hESC n = 3, CRISPR n = 6, Mutation n = 6). Data are shown as mean ± SD.*p < 0.05; data were compared by two-sample t-test. **E** Morphology of patient PBMCs and established LQT7 patient-derived iPSCs. **F** iPSCs from the mutation and CRISPR groups had a normal karyotype (Mutation n = 3, CRISPR n = 3). **G** iPSCs were stained for pluripotency markers (SSEA-4 and TRA-1-60) by flow cytometry analyzed (Mutation n = 6, CRISPR n = 6). **H** All iPSCs of the two groups gave rise to typical teratomas, which contained differentiated structures representing the three germ layers (nervous tissue [ectoderm], cartilage tissue [mesoderm] and intestinal epithelium [endoderm]) (Mutation n = 3, CRISPR n = 3)

The proband was a 21-year-old female. The pregnancy, delivery, and psychomotor development of her mother were described as normal. She experienced the first attack of lower limb weakness at the age of nine. She had presented with recurrent transitory episodes of weakness of the upper and lower limbs and obvious palpitation symptoms since then, especially after strenuous exercise. She was diagnosed with frequent ventricular arrhythmias when she was 15 years old. Recently, the attacks became more frequent and severe. She consulted our hospital when she showed long-term exercise and cold temperature as potential triggers for generalized paralysis accompanied by breathing difficulties. The attacks caused difficulties in walking and climbing steps lasting for approximately one week. Her main clinical manifestation during the episode was tetraplegia (level 4 on the MRC scale in the upper limbs and level 3 in the lower limbs), and she was asymptomatic during the interictal period. Examination showed neck fins, small hands and feet, short pinky on both sides, and fifth toe curvature. The limbs felt normal, and deep tendon reflexes were symmetric. There was no clinical myotonia. She had not experienced syncope before. Serum potassium levels were normal multiple times, only once dropping to 2.9 mEq/L. Serum creatine kinase levels during the episode were mildly elevated to 651 U/L (normal range: 24–195 U/L). Thyroid function and parathyroid hormone were normal. Electrocardiography (ECG) showed a U wave (Fig. 1A), and the corrected QT interval was normal (438 ms). Other ECGs showed frequent episodes of premature ventricular contraction (bipartite law, Fig. 1A). Moreover, she came from a family with a history of long QT syndrome. Her mother was diagnosed with hypokalemia cycle paralysis at the age of 13 years, accompanied by ventricular premature beats (QTc 578 ms), and her cousin received an implantable cardioverter defibrillator implant due to repeated loss of consciousness and prolonged QT interval. We sequenced the whole exomes of the family members of the patient in this study. The proband (IV-2) and the proband's mother (III-3), aunt (III-1) and cousin (IV-1) carried a heterozygous mutation in the *KCNJ2* gene at c.199C > T (p. R67W) with hypokalemia and ventricular arrhythmia symptoms (Fig. 1B), which was verified by Sanger sequencing (Fig. 1C). No other pathogenic mutations were detected in sarcomere-, ion channel-, cardiomyopathy- or other long QT syndrome-related genes, and the proband’s father (III-4) and uncle (III-2) carried no mutations in the *KCNJ2* gene (Fig. 1B). Combined with clinical symptoms (periodic paralysis, ventricular arrhythmia, abnormal appearance) and the genetic diagnosis, the diagnosis was ATS based on the criteria of the HRS/EHRA/APHRS expert consensus [22].

### Patient-derived iPSCs and CRISPR/Cas9‑mediated targeted gene correction

We successfully extracted and cultured iPSC lines from peripheral blood mononuclear cells (PBMCs) obtained from patients with LQT7, and constructed sgRNA to induce double-strand breaks near the mutation site to promote gene repair via homology-directed repair. Selecting monoclonal cell lines after amplifying for identification by first-generation sequencing, CRISPR clone cell lines with successful repair were obtained. Subsequently, we utilized stable iPSC lines past the 20th generation with a typical iPSC morphology (Fig. 1E). Upon quantification from at least three independent differentiations for each hiPSC line, all tested iPSC lines exhibited expression levels of endogenous pluripotency-related genes (*POU5F1* and *NODAL*) similar to those observed in hESCs (Fig. 1D). Additionally, the iPSCs showed long telomeres and normal karyotypes (Fig. 1F) and stained for pluripotency markers (SSEA-4 and TRA-1-60) by flow cytometry (Fig. 1G). These iPSC lines produced teratomas following subcutaneous injection in SCID mice, and all teratomas were shown differentiation of 3 embryonic germ layers (glandular structures, cartilage and neuroepithelium) (Fig. 1H). All animal experiments were performed in accordance with the guidelines from NIH Guide for the Care and Use of Laboratory Animals and approved by the Animal Ethics Committee of the Peking Union Medical College Hospital. To reduce the stress and pain of the mice, we euthanized them by cervical dislocation after the experiment.

### CM differentiation from iPSCs

**Fig. 2:**
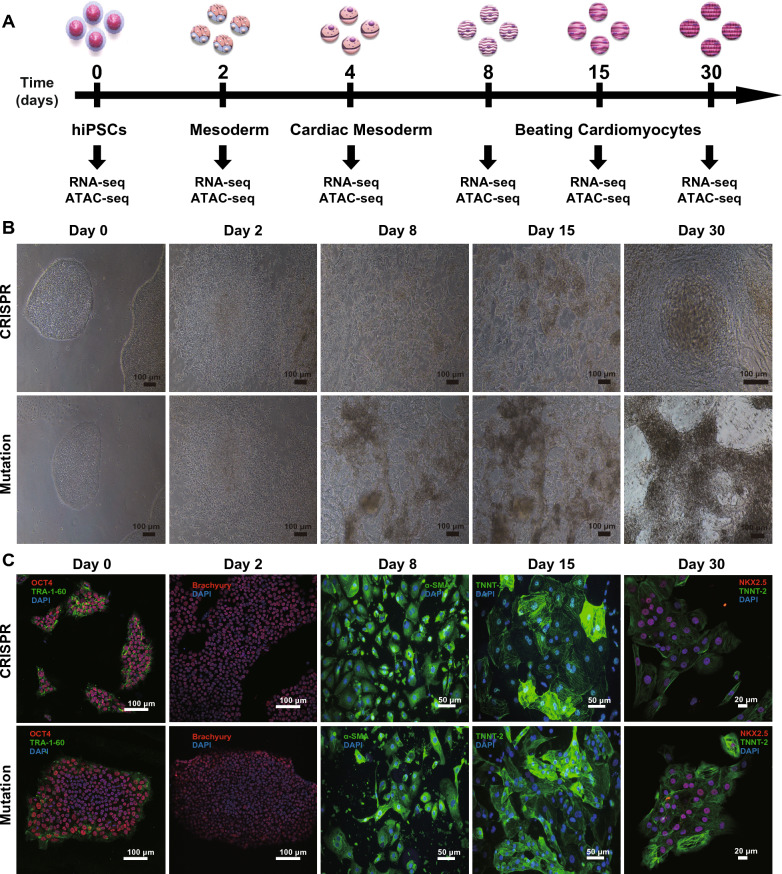
Identification of hiPSC-derived CMs at different stages of differentiation in the CRISPR and mutation groups. **A** Schematic showing the experimental design used for cardiomyocyte differentiation and sample collection. Cells at different stages of differentiation were collected on days 0, 2, 4, 8, 15 and 30 for RNA-seq and ATAC-seq (Mutation n = 12, CRISPR n = 12). **B** Morphology of the cells at different stages of differentiation. On the 8th day, cardiomyocytes with local spontaneous beating appeared. **C** Immunostaining of cells with specific markers corresponding to each time stage, including TRA-1-160 (green) and OCT4 (red) for hiPSCs (day 0), Brachyury (red) for the mesoderm (day 2), α-SMA (green) and NKX2-5 (green) for days 8 and 15, and NKX2-5 (red) and TNNT2 (green) for cardiomyocytes (day 30). The nuclei were stained with DAPI (blue)

With a well-developed method described in our previous study [12], we used a monolayer differentiation method to reproducibly generate CMs from the three hiPSC lines (mutation group, matched control group and CRISPR group). This method [23, 24] usually proceeds through six different stages before more mature CMs are obtained: iPSCs (day 0), mesoderm (day 2), cardiac mesoderm (day 4), early beating CMs (day 8), CMs with stable electrophysiological characteristics (day 15), and mature CMs (day 30) (Fig. 2A). They stably produced approximately 90–95% CMs (Fig. 2C, day 30). All iPSCs uniformly expressed the pluripotency markers OCT4, stage-specific embryonic antigen-4 (SSEA-4), TRA-1-60, and SOX2 (Fig. 2C, day 0). After two days of differentiation, the cells began to express the typical mesoderm protein marker Brachyury (Fig. 2C, day 2). Myocardial cells could beat locally or even over a large range within 7 to 10 days after the start of differentiation (Online Videos I). Both NK2 homeobox 5 (NKX2-5) and Troponin T type 2, cardiac (TNNT2) were strongly expressed at day 30 (mature CMs, Fig. 2C). These results suggest that these two cell lines exhibited the same CM differentiation kinetics.

### Identification and electrophysiological description of single LQT7-iPSC-CMs

**Fig. 3:**
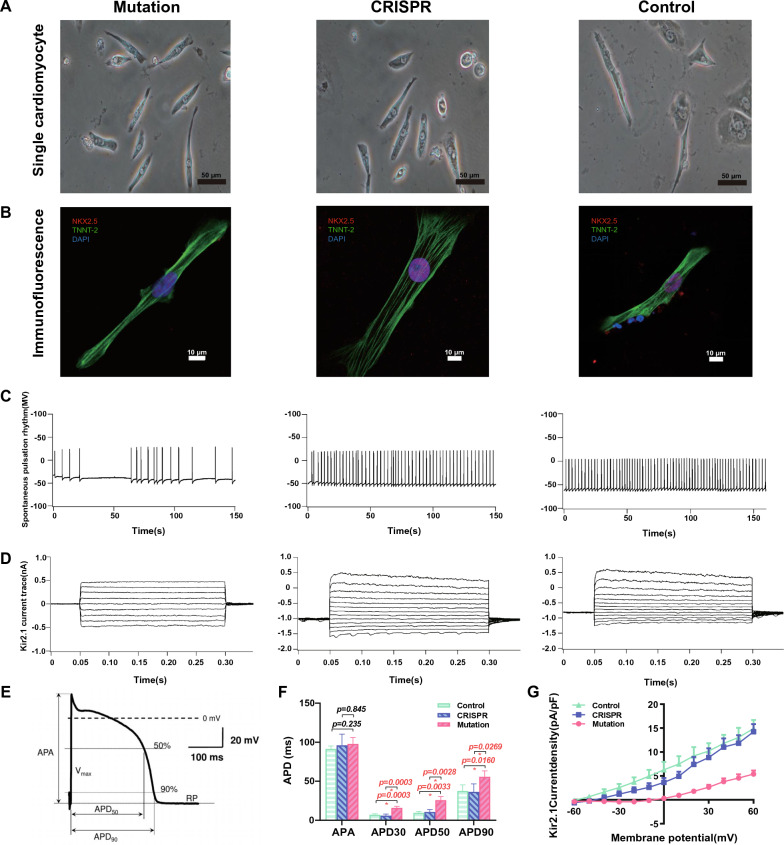
Morphology and immunofluorescence identification of single cardiomyocytes and electrophysiological characterization of LQT7-iPSC-CMs. **A** The morphologies of single CMs in the different groups were similar, and the CMs could undergo contraction independently, as observed by microscopy. **B** Immunofluorescence identification of single CMs based on NKX2-5 (red, in the nucleus) and TNNT2 (green, myofilament). **C** The rhythm of spontaneous contraction of single cells in the three groups. **D** Typical patch clamp images of Kir2.1 potassium ion channel current changes in the CMs of each group are shown. **E** Meaning of the action potential-related electrophysiological values APA, APD_30_, APD_50_ and APD_90_. **F** The action potential-related values of the CMs in each group were compared (Mutation n = 5, CRISPR n = 5, Control n = 5). **G** The potassium ion channel Kir2.1 is encoded by the *KCNJ2* gene. The figure shows its current trace and I–V curve in a voltage-clamp pattern resulting from a holding potential of − 60 mV and test pulses ranging from − 60 to + 60 mV (Mutation n = 5, CRISPR n = 5, Control n = 5). Data are shown as mean ± SD.*p < 0.05; data were compared by two-sample t-test

After CRISPR repaired of the *KCNJ2* gene, the morphology of single CMs in the CRISPR group was still similar to that in the mutation group. The CM morphology of both groups was also similar to that of the control group (taken from a healthy control population matched by age and sex) (Fig. 3A). The specific immunofluorescence identification of single iPSC-CMs with contractile autonomy in the three groups was consistent (Fig. 3B). Single-cell patch clamp action potentials and potassium Kir2.1 currents were measured in these three groups. All groups of single myocardial cells could produce spontaneous beats, of which the beats of the CRISPR group and healthy control group were regular, with approximately 30 beats/min in the CRISPR group and 35 beats/min in the control group. However, the beats of the mutation group were irregularly prolonged (Fig. 3C). Voltage clamp studies showed typical images of Kir2.1 current changes measured in the CMs of each group (Fig. 3D). When the action potential characteristics of each group were compared based on their electrophysiological characteristics (Fig. 3E), it was found that most action potential-related values (APD30, APD50 and APD90) of the mutation group were markedly prolonged from those of the CRISPR and healthy control groups and increased, except for APA (n = 10, Fig. 3F). The specific numerical mean ± standard deviation and P value of the mutation group (the former) and CRISPR group (the latter) were as follows: APD30 (15.91 ± 1.86 vs. 5.99 ± 1.99 ms, p = 3.48e−4), APD50 (25.65 ± 4.64 vs. 10.75 ± 2.92 ms, p = 2.75e−3), and APD90 (55.63 ± 7.64 vs. 37.54 ± 7.77 ms, p = 2.69e−2) (Fig. 3F). Additionally, the Kir2.1 current peak density of hiPSC-CMs in the mutation group was 5.39 ± 1.67 pA/pF, and 14.24 ± 3.6 pA/pF (mean ± SD, n = 8) in the CRISPR group (p = 2.99e−3, Fig. 3G), which showed that the amplitude of the inward rectified potassium ion current of CMs in the mutant group was significantly reduced. However, there was no significant difference between CRISPR group and the healthy control group (Fig. 3G).

### Differentiation stage-related modules identified by WGCNA and motif analysis

**Fig. 4:**
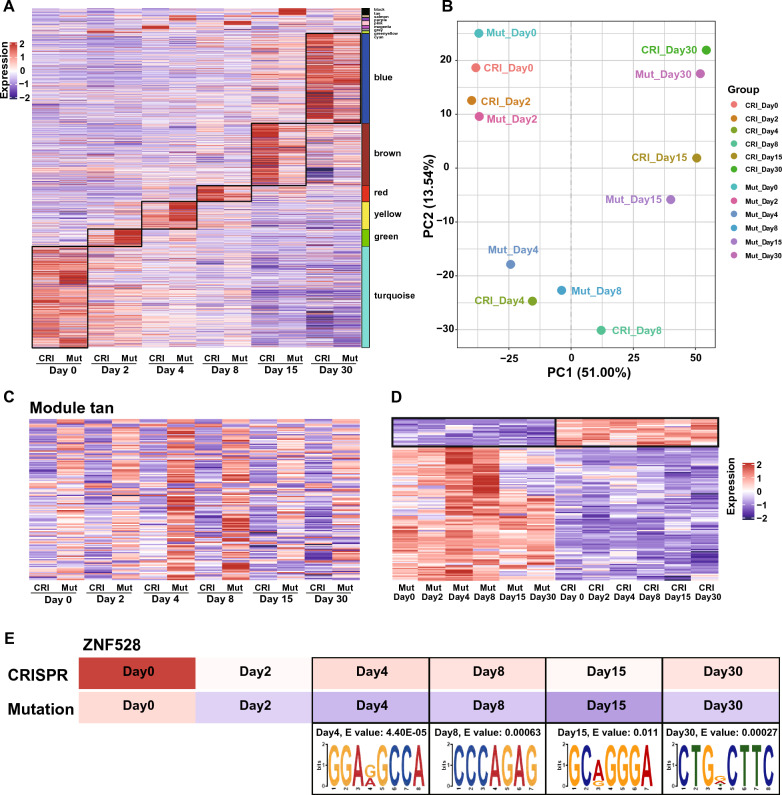
Differentiation stage-related modules identified by WGCNA and motif analysis. **A** Heatmap of gene expression profiles clustered by WGCNA. Six homogeneity aggregation modules with the same changes in expression over time in the cardiomyocyte differentiation stage were screened (Mutation n = 6, CRISPR n = 6). **B** PCA of the transcriptomes derived from the mutation and CRISPR groups during cardiomyocyte differentiation. **C** Expression heatmap for the tan module, which shows expression heterogeneity over time (Mutation n = 6, CRISPR n = 6). **D** The heatmap with low expression in the mutation group was explored for subsequent alignment to matched transcription factors (Mutation n = 6, CRISPR n = 6). **E** The transcription factor ZNF528 was obtained after alignment, and corresponded to the duration over which chromatin accessibility was high by verifying based on ATAC-seq data obtained in this study (Mutation n = 6, CRISPR n = 6)

To understand changes in gene expression during cardiac cell differentiation after derivation from iPSCs in the mutation and CRISPR groups, we performed RNA-Seq experiments on days 0, 2, 4, 8, 15 and 30, followed by clustering analysis, principal component analysis (PCA), and correlation analysis. A total of 21,292 genes were used for WGCNA after filtration (edgeR p adj < 0.05 |log2foldchange|> 1.0) based on the FPKM values, leading to 14 modules by coexpression patterns analysis (Fig. 4A). Subsequently, the significance of correlations between modules and factors was calculated based on their correlation with two factors: days and cell line. The 14 modules were the salmon, turquoise, green, green‒yellow, blue, cyan, black, brown, tan, purple, yellow, magenta, pink, red and gray modules. The gray module is an irregular expression module by default, so no follow-up analysis was carried out. Based on WGCNA clustering analysis, the expression of six modules showed highly correlated transcriptomic profiling and the same change over time among the two groups during different specific stages of CM differentiation, which called they homogeneity modules (Fig. 4A). These six modules enriched in a distinct array of biological functions (Table 1) and strongly correlated with CM differentiation stage. That is, in the GO enrichment analysis (Table 1), the pathways in which genes in the turquoise module were enriched at the stem cell stage (day 0) were mainly related to the mitotic cell cycle. The genes of the green module enriched at the mesoderm cell stage (day 2) were involved in the formation and development of the mesoderm. The pathways of the yellow module corresponded to the cardiac mesodermal precursor cell stage (day 4), which is mainly involved in the formation and development of embryonic organs in the late mesodermal stage, mainly the transformation of the mesoderm into cardiac muscle cells. The genes in the red module were expressed at the highest levels at the CM stage (day 8), when beating first occurs. At this time, these genes are not only affected by the regulation of stem cell proliferation but also related to heart formation. The brown module genes were mainly correlated with the translation of mitochondria, oxidative phosphorylation, ATP synthesis and metabolism at the electrophysiological mature CM stage (day 15), suggesting that CM growth and development require the consumption of a large amount of energy to maintain muscle contraction. When we obtained the blue module, corresponding to late CM stage (day 30), we found that the correlated pathways were mainly related to cardiac function, such as heart contraction, the regulation of heart rate, cardiac muscle cell action potential, cardiac conduction, and muscle tissue development. Expression of the genes in the above six modules was significantly increased in the specific corresponding period, which also proved that our process used to induce the directional differentiation of CMs was appropriate.

**Table 1:** GO terms significantly enriched in each module (adj. p < 0.05)

Time	Module	No. of genes	Adj. *p* value	GO terms (BP) ID	Description
Day0	Turquoise	3322	6.20e−08	GO:0044786	Cell cycle DNA replication
			3.22e−05	GO:0000075	Cell cycle checkpoint
			3.92e−08	GO:0000070	Mitotic sister chromatid segregation
			7.99e−09	GO:0140014	Mitotic nuclear division
			1.27e−10	GO:0071103	DNA conformation change
Day2	Green	482	0.011	GO:0048332	Mesoderm morphogenesis
			0.001	GO:0007498	Mesoderm development
			0.011	GO:0001707	Mesoderm formation
			2.82e−05	GO:0001756	Somitogenesis
			4.50e−06	GO:0009952	Anterior/posterior pattern specification
Day4	Yellow	685	0.011	GO:0048562	Embryonic organ morphogenesis
			0.026	GO:0048568	Embryonic organ development
Day8	Red	568	0.035	GO:0060914	Heart formation
			0.004	GO:0048562	Embryonic organ morphogenesis
			0.011	GO:0072091	Regulation of stem cell proliferation
Day15	Brown	2333	1.69e−07	GO:0140053	Mitochondrial gene expression
			4.64e−12	GO:0070125	Mitochondrial translational elongation
			2.00e−20	GO:0006119	Oxidative phosphorylation
			3.59e−17	GO:0042775	Mitochondrial ATP synthesis coupled electron transport
			1.10e−13	GO:0046034	ATP metabolic process
Day30	Blue	3064	1.39e−12	GO:0060047	Heart contraction
			6.11e−08	GO:0002027	Regulation of heart rate
			2.14e−07	GO:0086001	Cardiac muscle cell action potential
			1.41e−06	GO:0061337	Cardiac conduction
			5.45e−10	GO:0060537	Muscle tissue development

**Fig. 5:**
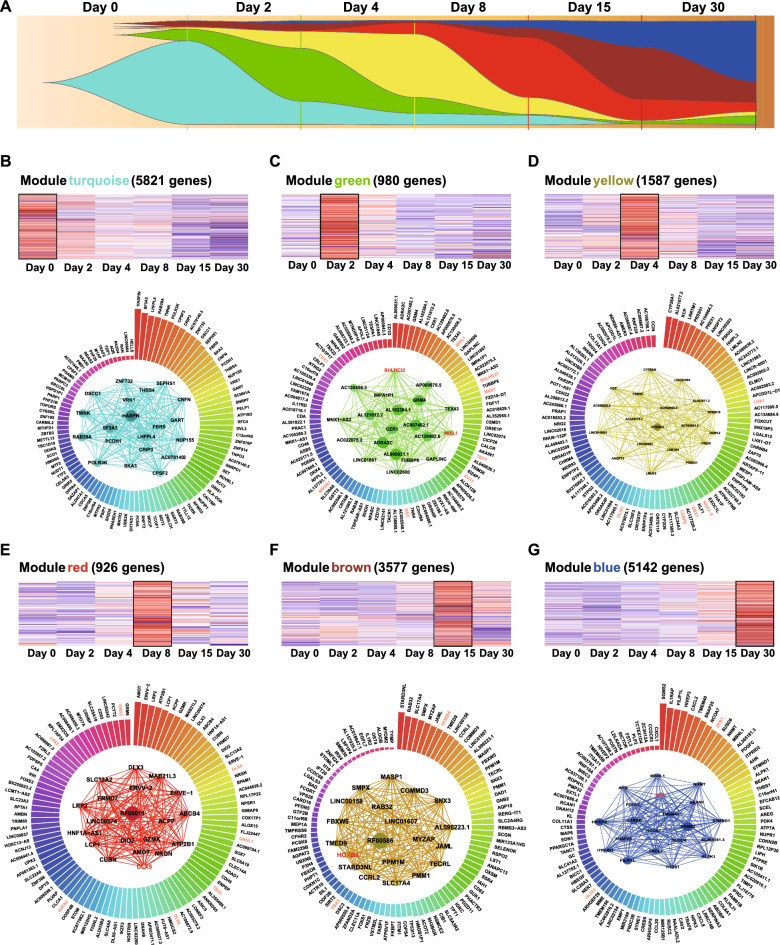
Screening target genes and transcription factors in the homogeneity module. **A** The fishplot shows the expression proportion (i.e., expression importance) of the six modules at different stages of differentiation and development. The abscissa represents time, and the ordinate represents the gene expression level. **B**–**G** The upper part shows a heatmap of the expression of genes in different modules at different periods, and the lower circular bar chart shows the top 100 genes with weighted importance and their rankings in each module. The mesh map shows the top 20 most important genes and their relationships in the module. The higher the weight is, the larger the icon, and the stronger the interaction, and the thicker the line. Red font indicates important TFs identified by alignment with human TF data from a public database (the JASPAR human database)

The segregation of samples by time point was also observed using PCA (Fig. 4B). The stem cell stage (day 0) and the cells at early induction (day 2) were similar, and samples on day 4 were similar to those on day 8, indicating that global gene expression was similar between the mesoderm stage and cardiac-mesoderm stage. The stage over which CMs gradually mature (day 30) could be clearly distinguished from the early CM stage (day 15). The target genes and transcription factors (TFs) were obtained by further analyzing the above genes with closely related to the development and differentiation of the CMs in the homogeneity module of two groups (Fig. 5). Next, we explored time-dependent expression changes to identify genes and TFs related to pathogenicity from the heterogeneous module. A heatmap comparing expression in the mutation group and CRISPR group showed temporal heterogeneous expression in only the tan module (Fig. 4C); that is, overall gene expression in the two groups of iPSC-CMs in this module was opposite that observed at the same period. The clustering heat map showed overall lower gene expression in the mutation group (Fig. 4D), which was consistent with the trend of KCNJ2 gene deletion or decreased function in LQT7 disease. Subsequently, referring to a public database of human TFs (the JASPAR human TF database) combined with the ATAC-seq data measured in our study, we found and verified that the expression of the corresponding TF ZNF528 was decreased in the mutation group with gradual myocardial development (expression changed in an opposite way in the CRISPR group, Fig. 4E). The results showed that the chromatin accessibility of ZNF528 began to increase on day 4 in the repaired CRISPR group and continued to change over time (Fig. 4E). The high level of accessibility of the mutation group decreased at the mature CM stage (Gradually changing from red to light purple in the Fig. 4E), and the expression of downstream target genes was then downregulated, indicating that this change in accessibility might be related to the pathogenicity of LQT7.

### Screening target genes and TFs in the homogeneity module

We synthesized the weighted importance scores and relevant connection information obtained by WGCNA, and created a fishplot showing the proportion and importance of module genes at different stages of CM development and differentiation. In a cohort analysis of myocardial differentiation stages, the proportion of genomic expression in the six modules varied over time (Fig. 5A). As shown in the figure, the expression of turquoise module genes was the highest on the 0th day of stem cells and continued until the mesoderm stage on the 2nd day, indicating that they were the important core genes at this stage. Additionally, the expression of green module genes began to gradually rise at day 2 and the expression of yellow module genes was the highest and most important on day 4. On days 8 and 15, the expression and importance of red module genes increased. The expression of brown module genes began to increase on day 15, but other modules genes were decreased. On the last day (day 30), the expression and proportion of blue module genes were increased and the largest. The heat maps of each module were then compared over time (Fig. 5B–G), and the above results were consistent with the expression changes over time revealed by the cluster heatmap (Fig. 4A). Specifically, the top 100 genes with the highest weight of each module were ranked and used to generate a circular histogram (Fig. 5B–G). The higher the weight, the higher the gene column, and the matching potential regulatory TFs (red font) were highlighted. In the middle of the histogram, a tight network of interactions is formed between the top 20 genes (the greater the weight, the greater the area of the dots. Figure 5–G). The number of matched potential regulatory TFs in modules were as follows: zero in turquoise (day 0), 8 in green (day 2), 5 in yellow (day 4), 7 in red (day 8), 2 in brown (day 15), and 2 in blue (day 30).

Based on the gene-enriched GO terms enriched (biological processes, BPs), the green module (Fig. 5C) was correlated with the mesoderm stage, which including development, morphogenesis and formation. Many known TFs assigned to this module play important roles in mesoderm, including mixed paired-like homeobox (*MIXL1*)*,* basic helix-loop-helix family member e22 (*BHLHE22)*, motor neuron and pancreas homeobox 1 (*MNX1*), T-box transcription factor T (*TBXT*), even-skipped homeobox 1 (*EVXT*), EVX1, goosecoid homeobox (*GSC*), mesogenin 1 (*MSGN1*) and caudal type homeobox 1 (*CDX1*). We also observed that two of the TFs (*MIXL1* and *BHLHE22*) exhibited highly weighted connectivity within the green module. The BPs of yellow and red modules included embryonic organ morphogenesis and development, indicating that these genes are related to the transition from the mesoderm to the early cardiac mesoderm. The yellow module (Fig. 5D) contained five potentials regulatory TFs with high weighted connectivity: LIM homeobox 1 (*LHX1*), NK2 homeobox 2 (*NKX2-2*), gastrulation brain homeobox 1 (*GBX1*), CCAAT enhancer-binding protein epsilon (*CEBPE*), and GLIS family zinc finger 1 (*GLIS1*). The red module (Fig. 5E) was related to the postcardiac-mesoderm stage and contained 7 assigned TFs: distal-less homeobox 5 (*DLX5*), grainyhead like transcription factor 1 (*GRHL1*), T-box transcription factor 3 (TBX3), distal-less homeobox 6 (*DLX6*), GATA binding protein 2 (*GATA2*), LIM homeobox 5 (*LHX5*), and empty spiracles homeobox 2 (*EMX2*). The brown (Fig. 5F) and blue modules (Fig. 5G) contained a total of four TFs, namely, homeobox B4 (*HOXB4*), T-box transcription factor 5 (*TBX5*), zinc finger E-box binding homeobox 1 (*ZEB1*) and GLIS family zinc finger 3 (*GLIS3*). All of these TFs were temporally overexpressed at the corresponding stages. The brown module was related to mitochondria and energy metabolism pathways, and the blue module was related to the regulation of heart and cardiac muscle contraction, development, action potential, and other functions of CM structure. These matched TFs were further verified by ATAC-seq experiment data.

### Dynamics of chromatin accessibility during CM differentiation in the mutation and CRISPR groups

**Fig. 6:**
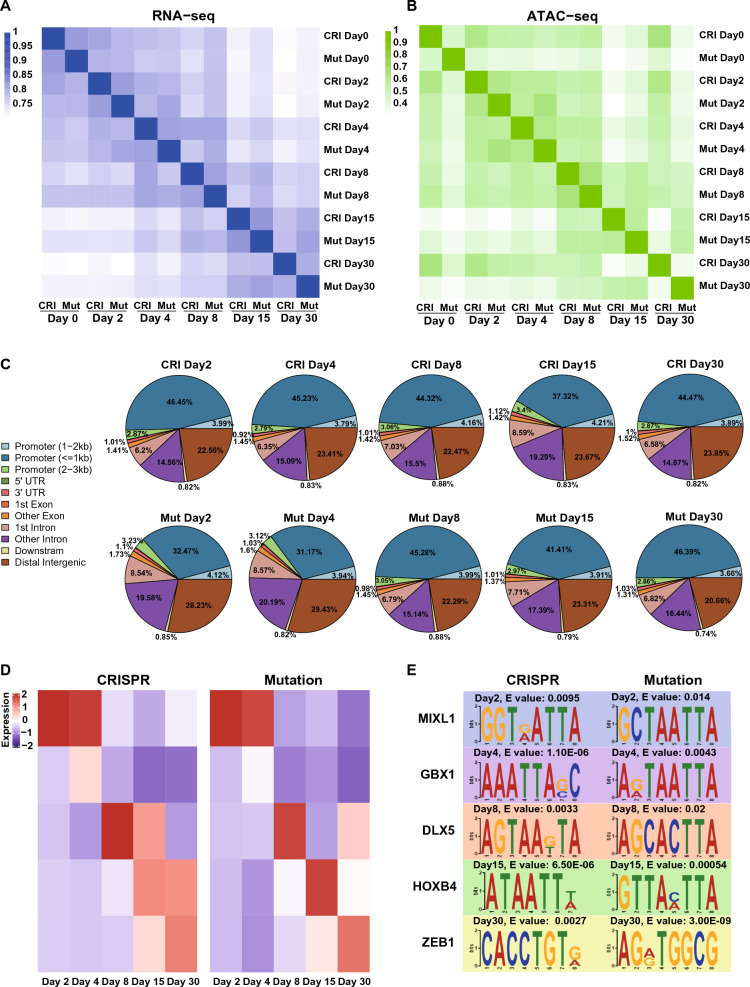
Dynamics of chromatin accessibility during cardiomyocyte differentiation in the mutation and CRISPR groups. **A**, **B** Correlation analysis of the RNA-seq data and ATAC-seq data at different stages. The colors represent the Kendall correlation coefficients between samples, which were calculated using FPKM values for gene expression (for RNA-seq) and genome-wide chromatin accessibility (for ATAC-seq). The darker the color is, the higher the correlation. **C** Changes in overlapping open chromatin regions between different groups of cells and cell lines at different stages. **D** Motif analysis: matched motifs from the differential peaks in the ATAC-seq data from the mutation and CRISPR groups are shown. **E** Verification to determine whether the gene expression of the corresponding transcription factor in a specific time period correlates with an open chromatin state. The E-value is the enrichment p-value times the number of candidate motifs tested

Through Kendall cluster analysis, we observed both similarities and subtle differences between RNA-seq and ATAC-seq data (Fig. 6A and B) at Adjacent stages, indicating that the overall differences in gene expression and chromatin accessibility between the two groups were closely related to the stages of CMs development and differentiation. The high comparative consistency of sample clustering patterns of RNA-seq data suggested that the two groups were highly similar in transcriptional regulation at each stage. Although the ATAC-seq data from cells in the mutation group on day 0 were slightly less correlated, the change in chromosome accessibility on day 2 (iPSC stage), day 4 (cardiac mesodermal cell stage) and day 8 (early beating CM stage) in the mutation group and repair CRISPR group were similar and obviously clustered together, suggesting a tight transition from iPSCs to early CMs. In addition, the ATAC-seq data from the two groups on day 15 (early electrophysiologically mature CM) and day 30 (late CMs) were significantly different, indicating that the function and regulation had changed at the mature CM stage. We used ChiPseeker software to calculate the distribution of peaks of ATAC-seq data for various functional areas collected at different stages, and it showed changes in chromosome accessibility during the differentiation of CMs in the mutation group and CRISPR group. The accessibility of the promoter region increased at the developing CMs stages of two groups, fluctuating between 44 and 52% (Fig. 6C), indicating an increase in transcription events.

GO enrichment analysis of consensus module genes in the two groups showed that they underwent a similar change from the whole process of CMs differentiation (Fig. 6D). Additionally, we obtained the potential pathogenicity TFs from the above results and verified them with our ATAC-seq data. Detect the open alignment of transcription factors in the experimental samples, where the E-value of GBX1 in the yellow module (Day 4) was the minimum value in the important TFs of that module, and was therefore listed as the subsequent analysis object (Fig. 6E). The minimum E-value ranking of the important TFs in other modules were consistent with the weighted importance ranking, such as MIXL1 in the green module (Day 2), DLX5 in the red module (Day 8), HOXB4 in the brown module (Day 15), and ZEB1 in the blue module (Day 15) (Fig. 6E). Then, the following five consistently highly accessible TFs (Fig. 6E) were verified by detection: mesoderm (day 2, MIXL1), cardiac mesoderm cells (day 4, GBX1), immature myocardium (day 8, DLX5), early electrophysiologically mature myocardium (day 15, HOXB4), and late myocardial cells (day 30, ZEB1).

### Identification and verification key target proteins in CMs in the mutation and CRISPR groups

**Fig. 7:**
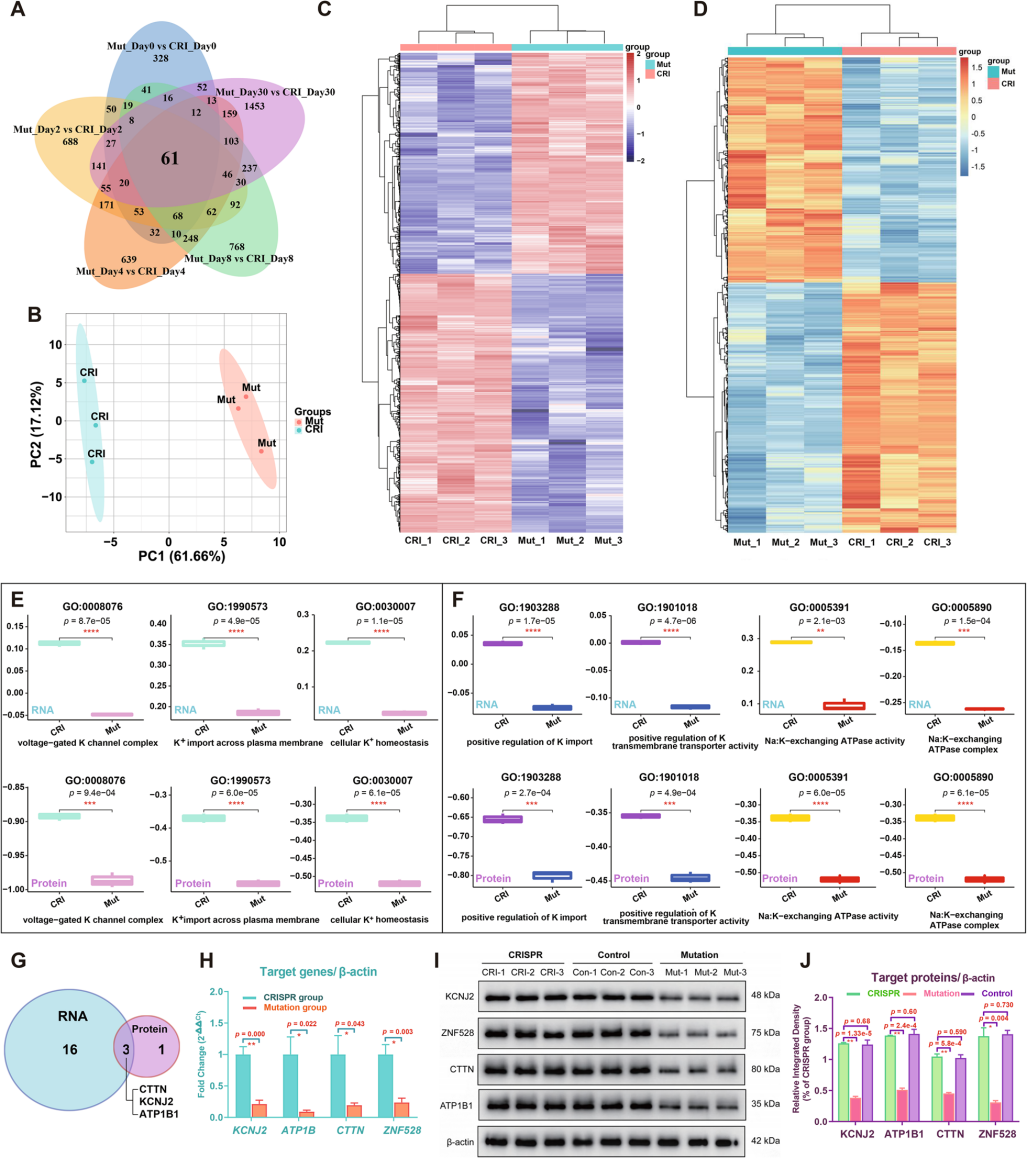
Expression analysis of potassium pathway-related genes and proteins in iPSC-CMs. **A** Venn diagram showing differentially expressed genes between the two groups at the same stages. **B** PCA scores from proteome-seq data for late iPSC-CMs (day 30) in two groups. Blue, CRISPR group; orange, mutation group. Heatmap analysis for the transcriptome (**C**) and proteome (**D**) (Mutation n = 3, CRISPR n = 3). **E** Comparison for the overall regulatory status of potassium channel-related pathways (including the participation of KCNJ2) at the gene expression level (upper row) and protein expression level (lower row) in the two groups (Mutation n = 3, CRISPR n = 3). **F** The overall regulatory status of four potassium ion-related pathways in CMs of the mutant group were significantly downregulated after comparison (Mutation n = 3, CRISPR n = 3). **G** A total of 3 target genes and their protein (CTTN, KCNJ2, ATP1B1) expression were consistently decreased in the mutant group by pathway expression analysis in a Venn diagram. **H** qRT‒PCR was performed to assess the expression of the target genes *KCNJ2*, *ATP1B1*, *CTTN*, and *ZNF528*, with β-actin used as a housekeeping gene (Mutation n = 6, CRISPR n = 6). **I**, **J** Detection of target proteins in iPSC-CMs (day 30) of the three groups (mutation, CRISPR, healthy control) by Western blotting (Mutation n = 3, CRISPR n = 3, Control n = 3). β-Actin was used as a loading control. Bands were quantified with Image J software. One asterisk indicates *p* < 0.05, and two asterisks indicates* p* < 0.001 compared with the other groups. Data are shown as mean ± SD.*p < 0.05; data were compared by two-sample t-test

During the progression of CMs (day 4 to day 30) in the mutation group, ZNF528 with reduced accessibility was related to pathogenicity in heterogeneous modules and its continuous low expression might affect the downstream target genes associated with LQT7 symptoms. A total of 61 differentially expressed genes between the two groups at the same stages by using a Venn diagram (Fig. 7A), and only 38 genes left after excluding zero expression value (Additional file 2: Table S2). Because the characteristics of late CMs were closest to the clinical symptoms of LQT7, we collected advanced mature CM samples (day 30) from the two groups and combined transcriptome and proteome data to jointly analyze the overall regulation of potassium-related pathways. PCA could well distinguished between two groups by proteome sequencing data (Fig. 7B). DESeq differential analysis (adj. p < 0.05) obtained 6,175 differentially expressed genes (2,844 upregulated and 3,331 downregulated) and 1050 proteins (497 upregulated and 553 downregulated) in the mutation group, and they were drawn for similarity clustering heatmaps (Fig. 7C and D).

The symptoms of LQT7 are mainly related to the dysfunction of potassium ions and the inhibition of related pathways. The potassium ion regulatory pathway was screened through GO pathway analysis, and the enrichment scores for the ssGSEA pathways (i.e., the activation score minus the inhibition score) were determined in the two groups to identify the pathways for which gene and protein expression changed differences statistically significant at the same time. The results showed that the enrichment fractions of the seven potassium channel-related pathways in the mutation group and the CRISPR group were significantly decreased (p < 0.05), and the three of them containing KCNJ2 protein were the voltage-gated potassium channel complex (GO: 0008076), potassium ion import across plasma membrane (GO: 1990573), and cellular potassium ion homeostasis (GO:0030007) (Fig. 7E). The other four pathways were the positive regulation of population ion import (GO: 1903288), positive regulation of population ion transmission transporter activity (GO: 1901018), spectrum: population exchanging ATPase activity (GO: 0005391), and spectrum: population exchanging ATPase complex (GO: 0005890) (Fig. 7F). There were 19 downregulated differentially expressed genes in the above seven potassium ion-related pathways, and 10 of them play a role in multiple different pathways at the same time (Additional file 3: Table S3). At the protein level, 4 proteins were contained in different potassium ion-related pathways, and 3 of them simultaneously appeared in different pathways (Additional file 4: Table S4). Three consistently downregulated proteins were obtained at the intersection of a Venn diagram (Fig. 7G); that is, key target proteins that are substantially regulated by TFs were obtained (Table 2). In addition to the known pathogenic protein KCNJ2, the cortactin (CTTN) and ATPase Na + /K + transporting subunit beta 1 (ATP1B1) proteins were newly discovered.

**Table 2:** List of genes and proteins that were consistently downregulated

Key target genes/proteins	CRISPR group		Mutation group		Difference comparison	
	Gene expression	Protein expression	Gene expression	Protein expression	Genetic differences	Protein differences
	Mean ± SD	Mean ± SD	Mean ± SD	Mean ± SD	t-test	t-test
CTTN	4436.2 ± 70.0	3469.0 ± 158.3	3739.5 ± 150.5	2907.3 ± 20.7	6.6E−03	2.4E−02
KCNJ2	266.0 ± 29.5	470.3 ± 33.0	22.2 ± 2.0	32.0 ± 3.9	4.7E−03	1.7E−03
ATP1B1	3683.1 ± 80.8	3517.6 ± 202.0	1727.6 ± 37.2	2851.8 ± 115.4	6.7E−05	1.4E−02

**Fig. 8:**
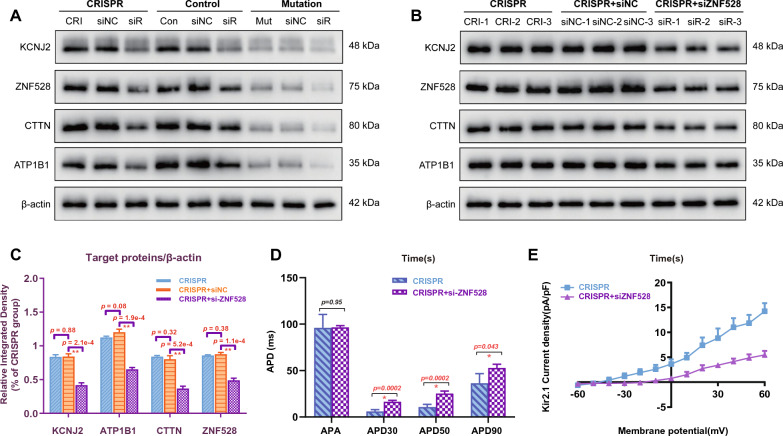
Quantification of their protein levels and electrophysiological characterization in hiPSC-derived cardiomyocytes treated with small interfering ZNF528 (si-ZNF528). **A** Detection of target proteins in iPSC-CMs of the three groups (Mutation n = 3, CRISPR n = 3, Control n = 3) treated with small interfering ZNF528. **B**, **C** Quantitative protein analysis were performed after intervention with si-ZNF528 and blank control plasmid (si-NC) in the CRISPR group (CRISPR n = 3, CRISPR + siNC n = 3, CRISPR + siZNF528 n = 3). **D** The action potential-related values of the CMs in each group were compared (CRISPR n = 5, CRISPR + siZNF528 n = 5). **E** The potassium ion channel Kir2.1 current trace and I-V curve in a voltage-clamp pattern resulting from a holding potential of − 60 mV and test pulses ranging from -60 to + 60 mV (CRISPR n = 5, CRISPR + siZNF528 n = 5). β-Actin was used as a loading control. Bands were quantified with Image J software. One asterisk indicates p < 0.05, and two asterisks indicates p < 0.001 compared with the other groups. Data are shown as mean ± SD.*p < 0.05; data were compared by two-sample t-test

To further verify the expression of these key target proteins whose were regulated by the pathogenicity-related TF (ZNF528), we performed qPCR and western blot analysis to test their actual expression levels. The transcription of genes (*KCNJ2, ATP1B1, CTTN*, and *ZNF528*) of the mutation group was significantly reduced compared with that in CRISPR group (all p < 0.05, Fig. 7H). It showed that the mRNA level of *KCNJ2* in the mutation group was approximately one-fifth of those in the CRISPR group, and the other target genes (*ATP1B1*, *CTTN* and *ZNF528*) were expressed at relative proportions of 0.09, 0.19 and 0.24, respectively (Fig. 7H). Because the morphological and electrophysiological characteristics of myocardial cells in the repair group and the healthy control group were similar (Fig. 3), we also used the control group to explore the changes in the levels of these key target proteins. The levels of these proteins in the mutation group also changed significantly, and the levels of KCNJ2, ZNF528, CTTN and ATP1B1 were 0.30-, 0.23-, 0.43- and 0.37-fold, respectively, those of the CRISPR group (Fig. 7I, J). However, there was no significant difference in the expression levels of target proteins between the repair CRISPR group and the control group (all p > 0.05, Fig. 7I, J). To further explore and verify, we knocked down ZNF528 in hiPSC-derived cardiomyocytes using si-ZNF528, and the designed si-ZNF528 could effectively reduce the ZNF528 levels in three groups (Fig. 8A). The target proteins (KCNJ2, ATP1B1, CTTN and ZNF528) levels showed reduced significant changes (all p > 0.05, Fig. 8B, C) upon ZNF528 knockdown. The levels of KCNJ2, ZNF528, CTTN and ATP1B1 were 0.49-, 0.56-, 0.46- and 0.54-fold in CRISPR + siZNF528 group, respectively, compared with those of the CRISPR + siNC group (Fig. 8B, C). The most action potential-related values (APD30, APD50 and APD90) of the CRISPR + si-ZNF528 group were markedly prolonged and increased from those of the CRISPR group, except for APA (n = 10, Fig. 8D). The amplitude of the inward rectified potassium ion current of CMs showed reduction (Fig. 8E). These experimental results are strongly consistent with the results of integrative analysis described above, demonstrating the important roles of these target proteins and ZNF528 in the regulation of cardiac electrophysiological function in LQT7.

## Discussion

LQT7 is a rare cardiomyopathy with abnormal electrophysiology, and 70% of cases are related to the Kir2.1 inward rectifier potassium channel protein, which is encoded by the KCNJ2 gene, in ventricular muscle, skeletal muscle and brain tissue [25]. Previous studies in animal and drug-induced cell models have shown the importance of the *KCNJ2* gene, but they exhibit species differences or limitations. For example, whole-gene KCNJ2-knockout mice have a short survival time and there was no Kir2.1 current in their muscle cells [6, 26]. Although heterozygous Kir2.1−/ + mice exhibited a prolonged action potential duration and a significant increase in QRS and QT intervals, the heart rates of the control group and the disease group were consistent [27, 28], making the QT intervals of dependence on heart rates impossible to be observed. The canine CMs induced by cesium chloride or calcium chloride can successfully mimic the electrophysiological characteristics of LQT7 (a slightly prolonged QT interval and U wave) [9], but these primary cells cannot be passaged, exhibit poor repeatability. Furthermore, toad oocytes were used to imitate the electrophysiological characteristics of potassium channels [29], but they cannot autonomic contraction. However, the hiPSC-derived CM model used in this study avoids the above research problems and it can be further used to explore the developmental differentiation of LQT7 by combined with the different stages of CMs maturation.

In this study, we first compared the specific phenotypes and electrophysiological characteristics of hiPSC-CMs from LQT7 patients with mutation, matched healthy controls and cells after CRISPR-mediated mutation repaired. The successful reprogramming and directional induction of them provide a reliable platform for the exploration (Figs. 1 and 2). Compared with that in the control and CRISPR group, the action potential duration was significantly prolonged in the mutation group, and the inward current of the inward rectifier potassium channel was reduced. After the repair of the mutation site, the electrophysiological characteristics of the CRISPR group tended to be similar to those of the control group, and there was no significant difference in action potential duration or the results of inward rectifier potassium channel patch-clamp experiments between the two groups (Fig. 3). Quantitative detection of iPSC-CMs from LQT7 patients by patch-clamp experiments showed that the Kir2.1 current had a significant negative effect (Fig. 3). Not only were the important conserved genes and transcriptional regulators of myocardial development screened from the same modules (Figs. 4 and 5) but also the pathogenicity-related genes and TFs related to changes in chromatin accessibility were screened from the heterogeneous modules (Fig. 6) by WGCNA. Subsequently, ssGSEA was used to analyze overall potassium-related pathway regulation in mature CMs of the mutation group, and key genes and their encoded proteins were screened by with multiomics, with the results experimentally verified (Fig. 7). The purpose of the above steps was to clarify the molecular changes that occur in the early development and differentiation of CMs in LQT7 and identify key pathogenic molecules.

The results of GO pathway analysis of the six homogeneous modules obtained by WGCNA were consistent with the corresponding stages of CM development and differentiation (Fig. 5 and Table 2). Firstly, the pathways were enriched in the turquoise module were mainly related to mitosis, the cell cycle and stem cell self-renewal. Secondly, the pathways involved at the mesodermal cell stage (green module) were enriched in the formation and development of the mesoderm. Then, the pathways of the cardiac mesodermal precursor cell stage that were enriched in the yellow module were related to the formation and development of embryonic organs in the late mesoderm, and in early pulsating CMs that were enriched in genes in the red module were related to heart formation. In addition, the electrophysiologically mature CM phase (brown module) was mainly related to the processes of mitochondrial ATP synthesis and metabolism, which is consistent with the notion that CMs need to consume a great deal of energy at this stage to grow and develop while maintaining contraction. Finally, the blue module corresponded to the late CM phase of gradual maturation, and their enriched pathways were all related to cardiac function (cardiac contraction, heart rate regulation, action potential regulation, cardiac conduction, and muscle tissue development-related pathways). In addition, our results showed that the TFs matched to the above different phases modules were representative of myocardial growth and development (Fig. 5), which further proved the reliability of our CMs model. For example, *MIXL1* is a key player in Wnt/TGF-β signaling in the early mesodermal differentiation of hESCs [30, 31]. BHLHE22 is a novel regulator that can direct neural circuit assembly in part through precise regulation [32, 33]. Previous studies have confirmed that *LHX1* originates from the endoderm and plays an important role in development [34, 35], and *NKX2* genes are primarily expressed in the mesendoderm-derived organs, such as the heart and gut[36]. The *DLX* gene provides insight into vertebrate genomics and craniofacial evolution [37, 38], so it was speculated that the craniofacial dysplasia observed in this ATS patients might be related to abnormalities in *DLX* 5 and 6. GRHL1 is a conserved transcriptional regulator and key to vertebrate desmosome formation and development [39, 40]. Embryonic TBX3 form the mature cardiac conduction system [41], and it also as a key sinoatrial node TF for controlling the development and function of pacemaker[42, 43]. GATA2 negatively regulates cardiac differentiation at the mesoderm specification stage [44]. The changes of the above TFs could affect cardiac dysplasia. *HOXB4* regulates endothelial cell turnover and development [45]. TBX5 is a key regulator of heart development [46], and TBX5-dependent pathways are associated with heart development, CM function, and congenital heart disease [47]. Preclinical evidence has reported the therapeutic value of TBX5 protein in regulating adult arrhythmias [48]. *ZEB1* is functionally important for early cardiac differentiation [49] and to be a regulator of endothelial-mesenchymal transition during development [50]. *GLIS3* can direct the differentiation of hESCs into posterior neural progenitor cells [51]. These results suggested that cells of different stages could through plasticity regulation and ultimately differentiate into the same cell type (CMs in this study).

The ATAC-seq data showed that the accessibility of the corresponding matching TFs to chromatin decreased at the different stages (Fig. 6D and E); these TFs included MIXL1 (mesoderm stage), GBX1 (cardiac mesodermal precursor cell stage), DLX5 (immature myocardium stage), HOXB4 (electrophysiological mature myocardium stage) and ZEB1 (late myocardium stage). MIXL1 is expressed in the early differentiation of human PSCs toward cardiac mesodermal derivatives [52]. GBX1 is involved in the development of neurons in the dorsal and ventral spinal cord during embryogenesis [53], and DLX5 is involved in brain development [38]. High HOXB4 expression can enhance the differentiation of embryonic stem cells [54], and HOXB4 is an effective differentiation-promoting transcriptional regulator [55]. ZEB1 is an important regulator of early cardiac differentiation, especially in the mesoderm stage of the heart [49]. These changes reflect the features of cardiac differentiation. The chromatin accessibilities of the TFs HOXB4 (day 15) and ZEB1 (day 30) were increased in homologous modules, indicating that the function and regulatory mechanism of CMs at the stage changed from electrophysiological maturity to late functional maturity (Fig. 6D and E). Only pathogenicity-related TF ZNF528 was screened from the heterogeneous tan module. It belongs to the family of zinc finger adjacent-binding domains and is a potential pathogenic gene for neurodevelopmental disorders [56]. No research has proven the role of the TF ZNF528 in regulating cardiovascular disease, but we verified its pathogenicity from heterogeneity module in this study. ZNF528 began to exhibit chromatin accessibility on day 4 in the CRISPR group, and this accessibility continued to increase over time, while access was reduced at the same time in the mutation group. These results suggest that low ZNF528 expression may be related to the clinical symptoms of LQT7.

In order to further screen out key disease target proteins affected by ZNF528, we jointly analyzed the overall regulatory status of potassium ion-related pathways at the gene and protein levels. The results showed that three inhibited potassium channel-related pathways containing KCNJ2 were related to the whole process by which a potassium ion enters the inward rectifier potassium channel (p < 0.05, Fig. 7E and F). There were the potassium ion enters the cell to exert its effect, which is sensed by the voltage-gated potassium channel complex (GO: 0008076), the potassium ion enters the cell through the membrane (GO: 1990573), and finally, the homeostasis of the potassium ion in the cell is maintained (GO: 030007). This further indicated that expression of these proteins of LQT7 patients was downregulated during this process, which led to the clinical manifestation of QT interval prolongation. The other four downregulated pathways were related to the regulation of potassium transmembrane channels and sodium potassium exchange ATPase, suggesting that the protein or energy demand for regulating potassium influx may be indirectly affected by mutant gene *KCNJ2*, thus further aggravating the clinical symptoms. Subsequently, three key sustainably down-regulated target proteins (KCNJ2, CTTN and ATP1B1) were screened in the disease group. KCNJ2 is a known pathogenic mutant protein in LQT7 that was identified in this study, indicating the reliability of our methodology. Therefore, the newly identified CTTN and ATP1B1 proteins may also be related to LQT7 symptoms. CTTN is the actin-binding protein cortactin, and maintains the normal operation of the Kv1.5 channel (delayed rectifier K + current is rapidly activated by atrial muscle) in Purkinje fibroblasts [57]. Decreased expression of CTTN leads to arrhythmias in mice [58], so it may inhibit the potassium ion channel Kv1.5 in LQT7 patients for prolonging the APD and QT intervals to aggravate the clinical symptoms. ATP1B1 is a sodium potassium pump β type 1 protein by obtaining energy from ATP under normal conditions, and its low expression leads to cardiac systolic dysfunction in mice [59]. The decreased expression of the ATP1B1 protein in the mutation group may have prevented K + from being transferred into the cells through the sodium potassium pump, causing the total amount of potassium to decrease, which may aggravate the QT interval extension and abnormal cardiac contraction in LQT7 patients. Finally, the low expression changes in gene and protein levels of these three key targets and one TF were validated in newly collected advanced CMs from the disease group (Fig. 7H–J).

## Conclusion

Our study demonstrated that iPSC-CMs originating from LQT7 patients exhibited specific disease phenotypes, and the ZNF528 was linked to the pathogenicity. However, this observation needs to be confirmed by TF intervention, which might revert the iPSC-CM phenotype to normal. Also, although iPSC-CMs retain the genetic information of the patient and exclude the influence of environmental factors on the disease, possible differentiation of cardiac myocytes in the human body should also be considered. Overall, we verified the phenotypes and electrophysiological function of hiPSC-CMs from LQT7 patients, and the established a human CMs model of specifically reproduces the LQT7 symptoms for providing a reliable platform to explore the mechanism of this disease or potential drugs. Meanwhile, the TFs related to different development and differentiation stages and the pathogenic TF ZNF528 of LQT7 were screened. In the mutation group, multiple potassium ion-related pathways were inhibited, and three key target proteins (KCNJ2, CTTN and ATP1B1) were identified and verified by experiments. In the future, it is expected that intervening ZNF528 and these target proteins will help to improve the LQT7 symptoms of myocardial electrophysiological disorder. We hope to obtain a treatment that does not rely on gene editing and provides new ideas for the treatment of rare diseases.

### Supplementary Information

**Additional file 1: Table S1.** Primers for qRT-PCR.

**Additional file 2: Table S2.** Differentially expressed genes between the two groups at the same stages.

**Additional file 3: Table S3.** List of differentially expressed genes down regulated in potassium related pathways with significant differences.

**Additional file 4: Table S4.** List of down regulated differential proteins in differential potassium related pathways.

## Data Availability

The data underlying this article will be shared on reasonable request to the corresponding author.
