# Supplementary material for: Transcriptome and open chromatin analysis reveals the process of myocardial cell development and key pathogenic target proteins in Long QT syndrome type 7
Source: J Transl Med. 2024 Mar 25;22:307. doi: 10.1186/s12967-024-05125-7 (PMC10964537; doi:10.1186/s12967-024-05125-7)
Supplement: Supplementary file 1 — Additional file 1: Table S1. Primers for qRT-PCR. [file 12967_2024_5125_MOESM1_ESM.doc]

**Table** S1. Primers for qRT-PCR

| **Gene** | **Forward (5’ to 3’)** | **Reverse (5’ to 3’)** |
| --- | --- | --- |
| *KCNJ2* | CCAAAGCAGAAGCACTGGAG | AATCAAATACCCAACCAAGGC |
| *CTTN* | TCATTGCTCATTGTGGTAAAGC | GTGGGCGGTGTGTCTTTC |
| *ATP1B1* | ATGTGCCCAGTGAACCGAAA | TCCAGAGCAATTTCCCAGCC |
| *ZNF528* | CAGGGAGTGCATCAAAGGTGTG | GCTTGAAATAGCTGTAGATCACTC |
| si-ZNF528 | GUUCAAAGCUUGUGAUACATT | UGUAUCACAAGCUUUGAACTT |
