# Supplementary material for: Transcriptome and open chromatin analysis reveals the process of myocardial cell development and key pathogenic target proteins in Long QT syndrome type 7
Source: J Transl Med. 2024 Mar 25;22:307. doi: 10.1186/s12967-024-05125-7 (PMC10964537; doi:10.1186/s12967-024-05125-7)
Supplement: Supplementary file 2 — Additional file 2: Table S2. Differentially expressed genes between the two groups at the same stages. [file 12967_2024_5125_MOESM2_ESM.doc]

**Table S2. Differentially expressed genes between the two groups at the same stages**

**(without zero expression value)**

| **Group** | **CRISPR** | | | | | | **Mutation** | | | | | |
| --- | --- | --- | --- | --- | --- | --- | --- | --- | --- | --- | --- | --- |
| **Gene** | **Day0** | **Day2** | **Day4** | **Day8** | **Day15** | **Day30** | **Day0** | **Day2** | **Day4** | **Day8** | **Day15** | **Day30** |
| AC004556.1 | 24.07 | 32.74 | 22.51 | 20.13 | 33.21 | 8.52 | 1.20 | 1.97 | 1.87 | 1.40 | 1.92 | 1.05 |
| AC079062.1 | 0.05 | 0.01 | 0.03 | 0.05 | 0.06 | 0.04 | 2.66 | 1.83 | 2.38 | 2.00 | 1.84 | 1.65 |
| AC112487.1 | 0.53 | 0.35 | 0.14 | 0.16 | 0.29 | 0.19 | 1.84 | 2.43 | 1.02 | 0.76 | 0.52 | 1.05 |
| AGAP12P | 0.02 | 0.05 | 0.04 | 0.09 | 0.05 | 0.07 | 0.27 | 0.27 | 0.44 | 0.61 | 0.60 | 0.36 |
| AMH | 3.99 | 4.69 | 9.36 | 4.63 | 1.79 | 1.35 | 0.76 | 0.98 | 1.92 | 0.76 | 0.37 | 0.30 |
| AP005018.2 | 0.16 | 0.07 | 0.03 | 0.03 | 0.30 | 0.27 | 2.48 | 2.29 | 3.44 | 16.29 | 8.05 | 9.73 |
| BX322639.1 | 3.46 | 3.62 | 1.80 | 1.38 | 0.30 | 0.29 | 0.17 | 0.14 | 0.17 | 0.13 | 0.07 | 0.05 |
| C9orf64 | 0.50 | 0.54 | 0.54 | 1.26 | 1.97 | 0.97 | 4.51 | 5.97 | 4.99 | 6.61 | 6.71 | 5.12 |
| CLEC18B | 2.47 | 2.88 | 3.02 | 2.52 | 3.62 | 4.20 | 0.18 | 0.19 | 0.15 | 0.12 | 1.16 | 0.92 |
| FAR2P1 | 0.45 | 0.19 | 0.14 | 0.07 | 0.11 | 0.18 | 1.88 | 1.40 | 2.94 | 2.25 | 1.34 | 1.24 |
| FRG1CP | 11.58 | 18.09 | 19.59 | 14.94 | 25.96 | 20.56 | 2.59 | 4.60 | 6.80 | 4.09 | 7.88 | 7.31 |
| HLA-A | 27.93 | 31.09 | 16.73 | 13.90 | 42.95 | 25.25 | 84.62 | 157.28 | 93.86 | 50.48 | 99.07 | 76.45 |
| HLA-C | 20.80 | 13.64 | 22.51 | 15.92 | 25.15 | 29.81 | 75.69 | 79.58 | 68.67 | 58.28 | 199.41 | 115.96 |
| IFIT1 | 1.45 | 0.74 | 0.90 | 2.00 | 12.20 | 3.26 | 15.23 | 26.81 | 14.88 | 12.15 | 57.38 | 9.66 |
| ISG15 | 18.51 | 12.27 | 10.87 | 20.32 | 55.16 | 18.43 | 65.01 | 94.98 | 88.16 | 72.04 | 528.58 | 60.89 |
| KLF2P1 | 1.07 | 0.44 | 0.41 | 0.26 | 0.42 | 0.23 | 4.91 | 2.82 | 8.96 | 6.00 | 2.27 | 3.16 |
| LINC00960 | 0.49 | 0.34 | 0.62 | 0.52 | 0.24 | 0.56 | 2.71 | 2.84 | 3.43 | 2.34 | 3.60 | 3.61 |
| MED15P9 | 0.04 | 0.01 | 0.02 | 0.02 | 0.08 | 0.04 | 0.89 | 0.85 | 1.86 | 0.97 | 0.41 | 0.30 |
| MEG3 | 1.59 | 0.84 | 2.22 | 1.92 | 4.45 | 4.08 | 41.93 | 21.24 | 38.24 | 28.16 | 31.34 | 34.42 |
| MEG8 | 0.03 | 0.02 | 0.04 | 0.08 | 0.04 | 0.07 | 2.33 | 0.60 | 0.66 | 0.55 | 0.46 | 0.48 |
| MEG9 | 0.38 | 0.22 | 1.14 | 0.13 | 0.52 | 0.76 | 5.28 | 1.86 | 4.90 | 2.18 | 1.32 | 2.52 |
| NLRP2 | 25.05 | 14.86 | 16.55 | 12.00 | 3.95 | 5.01 | 0.52 | 0.60 | 0.22 | 0.11 | 0.02 | 0.02 |
| OAS1 | 0.03 | 0.01 | 0.03 | 0.10 | 0.12 | 0.36 | 0.49 | 1.16 | 0.75 | 0.82 | 11.60 | 1.13 |
| OAS2 | 0.02 | 0.02 | 0.04 | 0.10 | 0.04 | 0.04 | 0.77 | 2.69 | 1.42 | 1.07 | 11.34 | 0.55 |
| PCDHA10 | 0.56 | 0.49 | 0.38 | 0.30 | 0.24 | 0.29 | 1.57 | 1.86 | 2.09 | 1.62 | 1.13 | 1.41 |
| PCDHB15 | 3.70 | 3.54 | 4.34 | 3.98 | 3.37 | 2.94 | 1.09 | 1.34 | 1.30 | 0.53 | 0.53 | 0.93 |
| POTEE | 0.03 | 0.03 | 0.01 | 0.01 | 0.01 | 0.05 | 0.47 | 0.34 | 0.92 | 0.54 | 0.38 | 0.47 |
| POTEF | 0.04 | 0.02 | 0.04 | 0.02 | 0.03 | 0.13 | 0.71 | 0.58 | 1.58 | 1.19 | 0.48 | 0.68 |
| RPS28 | 281.13 | 428.61 | 298.43 | 304.67 | 456.82 | 154.76 | 62.08 | 71.67 | 67.03 | 41.17 | 43.56 | 33.70 |
| RPS28P7 | 39.88 | 62.75 | 51.38 | 43.65 | 73.69 | 23.63 | 3143.50 | 3515.81 | 3297.79 | 2444.83 | 2543.18 | 1834.09 |
| TFAP2A | 0.15 | 0.18 | 8.20 | 9.45 | 0.01 | 4.16 | 0.01 | 0.01 | 0.03 | 3.45 | 6.84 | 0.68 |
| TMEM132C | 0.24 | 1.50 | 1.00 | 1.00 | 2.40 | 3.08 | 1.01 | 4.41 | 6.14 | 0.07 | 1.81 | 0.42 |
| ZNF528 | 2.04 | 2.86 | 3.26 | 3.07 | 1.49 | 2.24 | 0.18 | 0.32 | 0.51 | 0.54 | 0.42 | 0.76 |
| ZNF560 | 6.02 | 5.71 | 3.11 | 1.52 | 0.20 | 0.33 | 0.01 | 0.02 | 0.01 | 0.08 | 0.06 | 0.05 |
| ZNF626 | 2.42 | 2.86 | 2.96 | 3.09 | 2.70 | 1.96 | 0.77 | 1.02 | 1.20 | 1.01 | 0.73 | 0.66 |
| ZNF66 | 2.70 | 1.88 | 3.20 | 2.16 | 0.63 | 0.46 | 0.59 | 0.57 | 1.27 | 0.83 | 0.30 | 0.08 |
| ZNF682 | 3.38 | 2.77 | 3.24 | 3.41 | 1.65 | 0.98 | 0.01 | 0.01 | 0.05 | 0.07 | 0.20 | 0.23 |
| ZNF737 | 4.42 | 4.34 | 6.77 | 5.61 | 3.07 | 3.44 | 0.42 | 0.10 | 0.17 | 0.18 | 0.27 | 0.15 |
