# Supplementary material for: Transcriptome and open chromatin analysis reveals the process of myocardial cell development and key pathogenic target proteins in Long QT syndrome type 7
Source: J Transl Med. 2024 Mar 25;22:307. doi: 10.1186/s12967-024-05125-7 (PMC10964537; doi:10.1186/s12967-024-05125-7)
Supplement: Supplementary file 3 — Additional file 3: Table S3. List of differentially expressed genes down regulated in potassium related pathways with significant differences. [file 12967_2024_5125_MOESM3_ESM.doc]

**Table S3. List of differentially expressed genes down regulated in potassium**

related pathways with significant differences

| **Path ID** | **Differentially expressed genes** | **CRI_1** | **CRI_2** | **CRI_3** | **Mut_1** | **Mut_2** | **Mut_3** | **Expression state** |
| --- | --- | --- | --- | --- | --- | --- | --- | --- |
| **GO:0008076** | CTTN | 4362.8 | 4502.2 | 4443.5 | 3617.7 | 3907.8 | 3693.0 | down |
|  | KCNA2 | 39.1 | 40.3 | 30.3 | 18.0 | 16.0 | 10.9 | down |
|  | KCNC1 | 35.1 | 43.3 | 51.9 | 8.0 | 15.0 | 12.8 | down |
|  | KCNC4 | 190.6 | 211.5 | 206.5 | 153.3 | 160.5 | 169.7 | down |
|  | KCND3 | 23.1 | 17.1 | 26.4 | 6.0 | 3.0 | 6.9 | down |
|  | KCNG1 | 643.2 | 604.3 | 680.2 | 527.0 | 544.3 | 515.9 | down |
|  | KCNJ2 | 284.0 | 282.0 | 232.0 | 20.0 | 23.9 | 22.7 | down |
|  | KCNJ5 | 436.5 | 454.3 | 448.3 | 412.8 | 345.9 | 372.9 | down |
|  | KCNJ16 | 18.1 | 23.2 | 29.4 | 5.0 | 7.0 | 9.9 | down |
|  | KCNK2 | 67.2 | 56.4 | 66.6 | 35.1 | 40.9 | 36.5 | down |
|  | KCNMA1 | 1809.1 | 1749.5 | 1792.1 | 1069.0 | 1100.6 | 1147.2 | down |
|  | KCNQ2 | 469.6 | 599.3 | 518.7 | 377.7 | 289.1 | 314.7 | down |
|  | KCNQ3 | 189.6 | 187.3 | 174.2 | 99.2 | 83.7 | 88.8 | down |
|  | CNTNAP2 | 730.5 | 849.1 | 743.8 | 612.1 | 531.3 | 561.3 | down |
|  | KCNV1 | 107.4 | 93.7 | 95.9 | 6.0 | 8.0 | 17.8 | down |
|  | KCNQ5 | 306.0 | 328.3 | 328.9 | 159.3 | 158.5 | 183.5 | down |
| **GO:1990573** | ATP1B1 | 3774.8 | 3622.0 | 3652.6 | 1736.2 | 1686.8 | 1759.7 | down |
|  | KCNJ2 | 284.0 | 282.0 | 232.0 | 20.0 | 23.9 | 22.7 | down |
|  | KCNJ5 | 436.5 | 454.3 | 448.3 | 412.8 | 345.9 | 372.9 | down |
|  | KCNJ16 | 18.1 | 23.2 | 29.4 | 5.0 | 7.0 | 9.9 | down |
|  | KCNK5 | 133.5 | 169.2 | 129.2 | 73.1 | 59.8 | 76.9 | down |
| **GO:0030007** | ATP1B1 | 3774.8 | 3622.0 | 3652.6 | 1736.2 | 1686.8 | 1759.7 | down |
|  | KCNJ2 | 284.0 | 282.0 | 232.0 | 20.0 | 23.9 | 22.7 | down |
|  | KCNMA1 | 1809.1 | 1749.5 | 1792.1 | 1069.0 | 1100.6 | 1147.2 | down |
| **GO:1903288** | ATP1B1 | 3774.8 | 3621.9 | 3652.6 | 1736.2 | 1686.8 | 1759.7 | down |
|  | WNK2 | 977.3 | 1060.6 | 1102.1 | 816.5 | 763.6 | 805.9 | down |
| **GO:1901018** | ATP1B1 | 3774.8 | 3621.9 | 3652.6 | 1736.2 | 1686.8 | 1759.7 | down |
| **GO:0005391** | ATP1B1 | 3774.8 | 3621.9 | 3652.6 | 1736.2 | 1686.8 | 1759.7 | down |
| **GO:0005890** | ATP1B1 | 3774.8 | 3621.9 | 3652.6 | 1736.2 | 1686.8 | 1759.7 | down |
