# Supplementary material for: Transcriptome and open chromatin analysis reveals the process of myocardial cell development and key pathogenic target proteins in Long QT syndrome type 7
Source: J Transl Med. 2024 Mar 25;22:307. doi: 10.1186/s12967-024-05125-7 (PMC10964537; doi:10.1186/s12967-024-05125-7)
Supplement: Supplementary file 4 — Additional file 4: Table S4. List of down regulated differential proteins in differential potassium related pathways. [file 12967_2024_5125_MOESM4_ESM.doc]

**Table S4. List of down regulated differential proteins**

**in differential potassium related pathways**

| **Path ID** | **Differentially expressed proteins** | **CRI_1** | **CRI_2** | **CRI_3** | **Mut_1** | **Mut_2** | **Mut_3** | **Expression state** |
| --- | --- | --- | --- | --- | --- | --- | --- | --- |
| **GO:0008076** | CTTN | 3570.3 | 3286.6 | 3550.1 | 2899.2 | 2891.9 | 2930.8 | down |
|  | KCNJ2 | 486.0 | 432.3 | 492.5 | 36.1 | 28.3 | 31.5 | down |
| **GO:1990573** | ATP1A3 | 490.8 | 563.3 | 537.3 | 372.4 | 377.2 | 373.5 | down |
|  | ATP1B1 | 3703.7 | 3302.7 | 3546.3 | 2830.5 | 2976.4 | 2748.5 | down |
|  | KCNJ2 | 486.0 | 432.3 | 492.5 | 36.1 | 28.3 | 31.5 | down |
| **GO:0030007** | ATP1A3 | 490.8 | 563.3 | 537.3 | 372.4 | 377.2 | 373.5 | down |
|  | ATP1B1 | 3703.7 | 3302.7 | 3546.3 | 2830.5 | 2976.4 | 2748.5 | down |
|  | KCNJ2 | 486.0 | 432.3 | 492.5 | 36.1 | 28.3 | 31.5 | down |
| **GO:1903288** | ATP1B1 | 3703.7 | 3302.7 | 3546.3 | 2830.5 | 2976.4 | 2748.5 | down |
| **GO:1901018** | ATP1B1 | 3703.7 | 3302.7 | 3546.3 | 2830.5 | 2976.4 | 2748.5 | down |
| **GO:0005391** | ATP1A3 | 490.8 | 563.3 | 537.3 | 372.4 | 377.2 | 373.5 | down |
|  | ATP1B1 | 3703.7 | 3302.7 | 3546.3 | 2830.5 | 2976.4 | 2748.5 | down |
| **GO:0005890** | ATP1A3 | 490.8 | 563.3 | 537.3 | 372.4 | 377.2 | 373.5 | down |
|  | ATP1B1 | 3703.7 | 3302.7 | 3546.3 | 2830.5 | 2976.4 | 2748.5 | down |
